# Supplementary material for: Donor whole blood DNA methylation is not a strong predictor of acute graft versus host disease in unrelated donor allogeneic haematopoietic cell transplantation
Source: Front Genet. 2024 Apr 3;15:1242636. doi: 10.3389/fgene.2024.1242636 (PMC11021570; doi:10.3389/fgene.2024.1242636)
Supplement: Supplementary file 1 [file Table1.DOCX]

***Table 1: Discovery and validation cohort characteristics****. Characteristics of adult patients undergoing first allogeneic PB HCT for acute leukemia or MDS from an 8/8 HLA-matched unrelated donor between 2000-2016 with available donor blood samples, as reported to the CIBMTR. Restricted to Caucasian donors, myeloablative preparative regimens,* ***no ATG/Campath*** *and patients surviving >100 days with no aGVHD or those that developed grades III-IV aGVHD at any time post-HCT. Donors were matched between comparison groups based on sex and age by decade.*

|  | Discovery cohort | | | Validation cohort | | |
| --- | --- | --- | --- | --- | --- | --- |
|  | No aGVHD +  100-day survival | Grades III-IV aGVHD |  | No aGVHD +  100-day survival | Grades III-IV aGVHD |  |
| Variable | N (%) | N (%) | p-value^a^ | N (%) | N (%) | p-value^a^ |
| Number of Recipients | 141 | 141 |  | 144 | 144 |  |
| Disease |  |  | 0.339 |  |  | 0.020 |
| AML | 85 (60) | 73 (52) |  | 100 (69) | 77 (53) |  |
| ALL | 24 (17) | 31 (22) |  | 19 (13) | 27 (19) |  |
| MDS | 32 (23) | 37 (26) |  | 25 (17) | 40 (28) |  |
| Recipient Age |  |  | 0.464 |  |  | 1.000 |
| 18-29 | 28 (20) | 28 (20) |  | 18 (13) | 18 (13) |  |
| 30-39 | 26 (18) | 18 (13) |  | 24 (17) | 24 (17) |  |
| 40-49 | 36 (26) | 33 (23) |  | 34 (24) | 33 (23) |  |
| 50-59 | 33 (23) | 38 (27) |  | 39 (27) | 40 (28) |  |
| 60-69 | 15 (11) | 23 (16) |  | 28 (19) | 28 (19) |  |
| 70+ | 3 (2) | 1 (1) |  | 1 (1) | 1 (1) |  |
| Median (Range) | 45 (19-76) | 47 (18-72) | 0.541 | 49 (20-75) | 50 (19-71) | 0.998 |
| Recipient Sex |  |  | 0.716 |  |  | 1.000 |
| Male | 82 (58) | 85 (60) |  | 85 (59) | 85 (59) |  |
| Female | 59 (42) | 56 (40) |  | 59 (41) | 59 (41) |  |
| Recipient Race/Ethnicity |  |  | 0.113 |  |  | 1.000 |
| Caucasian | 134 (96) | 131 (94) |  | 144 (100) | 144 (100) |  |
| African American | 2 (1) | 1 (1) |  | 0 | 0 |  |
| Native American | 2 (1) | 0 |  | 0 | 0 |  |
| Caucasian, Hispanic | 2 (1) | 8 (6) |  | 0 | 0 |  |
| Unknown | 1 (N/A) | 1 (N/A) |  | 0 | 0 |  |
| Recipient ABO Type |  |  | 0.767 |  |  | 0.242 |
| A | 54 (45) | 52 (42) |  | 14 (40) | 24 (53) |  |
| B | 13 (11) | 15 (12) |  | 7 (20) | 3 (7) |  |
| AB | 3 (3) | 6 (5) |  | 4 (11) | 3 (7) |  |
| O | 50 (42) | 50 (41) |  | 10 (29) | 15 (33) |  |
| Unknown | 21 (N/A) | 18 (N/A) |  | 109 (N/A) | 99 (N/A) |  |
| Rh Factor |  |  | 0.788 |  |  | 0.167 |
| Positive | 105 (88) | 109 (89) |  | 33 (94) | 38 (84) |  |
| Negative | 15 (13) | 14 (11) |  | 2 (6) | 7 (16) |  |
| Unknown | 21 (N/A) | 18 (N/A) |  | 109 (N/A) | 99 (N/A) |  |
| Blood Type |  |  | 0.985 |  |  | 0.277 |
| A + | 46 (38) | 46 (37) |  | 14 (40) | 20 (44) |  |
| B + | 11 (9) | 13 (11) |  | 6 (17) | 3 (7) |  |
| AB + | 2 (2) | 4 (3) |  | 4 (11) | 2 (4) |  |
| O + | 46 (38) | 46 (37) |  | 9 (26) | 13 (29) |  |
| A - | 8 (7) | 6 (5) |  | 0 | 4 (9) |  |
| B - | 2 (2) | 2 (2) |  | 1 (3) | 0 |  |
| AB - | 1 (1) | 2 (2) |  | 0 | 1 (2) |  |
| O - | 4 (3) | 4 (3) |  | 1 (3) | 2 (4) |  |
| Unknown | 21 (N/A) | 18 (N/A) |  | 109 (N/A) | 99 (N/A) |  |
| Recipient CMV Status |  |  | 0.721 |  |  | 0.281 |
| Negative | 65 (46) | 65 (46) |  | 59 (41) | 69 (48) |  |
| Positive | 74 (52) | 74 (52) |  | 85 (59) | 74 (51) |  |
| Inconclusive | 1 (1) | 2 (1) |  | 0 | 1 (1) |  |
| Not tested | 1 (1) | 0 |  | 0 | 0 |  |
| Donor Age |  |  | 0.090 |  |  | 0.792 |
| 18-29 | 90 (64) | 76 (54) |  | 105 (73) | 103 (72) |  |
| 30-39 | 51 (36) | 65 (46) |  | 39 (27) | 41 (28) |  |
| Median (Range) | 26 (19-40) | 29 (19-40) | 0.003 | 26 (19-39) | 27 (19-39) | 0.076 |
| Donor Sex |  |  | 0.585 |  |  | 0.063 |
| Male | 103 (73) | 107 (76) |  | 98 (68) | 112 (78) |  |
| Female | 38 (27) | 34 (24) |  | 46 (32) | 32 (22) |  |
| Donor Race/Ethnicity |  |  | 1.000 |  |  | 1.000 |
| Caucasian | 141 (100) | 141 (100) |  | 144 (100) | 144 (100) |  |
| Donor ABO Type |  |  | 0.081 |  |  | 0.498 |
| A | 69 (49) | 51 (36) |  | 65 (45) | 60 (42) |  |
| B | 15 (11) | 14 (10) |  | 13 (9) | 8 (6) |  |
| AB | 6 (4) | 4 (3) |  | 6 (4) | 9 (6) |  |
| O | 51 (36) | 72 (51) |  | 60 (42) | 67 (47) |  |
| Donor CMV Status |  |  | 0.875 |  |  | 0.090 |
| Negative | 104 (74) | 105 (74) |  | 90 (63) | 104 (72) |  |
| Positive | 33 (23) | 32 (23) |  | 53 (37) | 38 (26) |  |
| Previously reported reactive | 1 (1) | 0 |  | 1 (1) | 0 |  |
| Not tested | 1 (1) | 1 (1) |  | 0 | 2 (1) |  |
| Unknown | 2 (1) | 3 (2) |  | 0 | 0 |  |
| Donor-Recipient Sex Match |  |  | 0.818 |  |  | 0.072 |
| Male-Male | 62 (44) | 69 (49) |  | 69 (48) | 69 (48) |  |
| Male-Female | 41 (29) | 38 (27) |  | 29 (20) | 43 (30) |  |
| Female-Male | 20 (14) | 16 (11) |  | 16 (11) | 16 (11) |  |
| Female-Female | 18 (13) | 18 (13) |  | 30 (21) | 16 (11) |  |
| Recipient Age at Diagnosis |  |  | 0.452 |  |  | 0.878 |
| < 10 | 0 | 1 (1) |  | 0 | 0 |  |
| 10-17 | 1 (1) | 4 (3) |  | 1 (1) | 0 |  |
| 18-29 | 29 (21) | 27 (19) |  | 20 (14) | 19 (13) |  |
| 30-39 | 29 (21) | 18 (13) |  | 23 (16) | 27 (19) |  |
| 40-49 | 31 (22) | 34 (24) |  | 34 (24) | 34 (24) |  |
| 50-59 | 36 (26) | 38 (27) |  | 39 (27) | 37 (26) |  |
| 60-69 | 13 (9) | 18 (13) |  | 26 (18) | 27 (19) |  |
| 70+ | 2 (1) | 1 (1) |  | 1 (1) | 0 |  |
| Median (Range) | 44 (16-75) | 46 (11-72) | 0.450 | 48 (17-75) | 49 (18-70) | 0.867 |
| MDS Disease Status |  |  | 0.465 |  |  | 0.941 |
| Early | 14 (44) | 13 (35) |  | 2 (8) | 3 (8) |  |
| Advanced | 18 (56) | 24 (65) |  | 23 (92) | 37 (93) |  |
| AML/ALL Disease Status |  |  | 0.137 |  |  | 0.009 |
| Early | 51 (47) | 43 (40) |  | 67 (56) | 67 (64) |  |
| Intermediate | 30 (28) | 21 (20) |  | 32 (27) | 11 (11) |  |
| Advanced | 23 (21) | 28 (27) |  | 20 (17) | 24 (23) |  |
| Unknown | 5 (5) | 12 (12) |  | 0 | 2 (2) |  |
| Conditioning Regimen |  |  | 0.114 |  |  | <0.001 |
| Bu + Cy | 49 (35) | 56 (40) |  | 53 (37) | 54 (38) |  |
| Bu + Mel | 1 (1) | 3 (2) |  | 0 | 0 |  |
| Bu + Flud | 38 (27) | 26 (18) |  | 61 (42) | 32 (22) |  |
| Mel + Flud | 0 | 1 (1) |  | 7 (5) | 10 (7) |  |
| Cy Alone | 43 (30) | 35 (24) |  | 22 (16) | 45(31) |  |
| Others | 10 (7) | 20 (14) |  | 1 (1) | 3 (2) |  |
| TBI Usage |  |  | 0.806 |  |  | 0.001 |
| Yes | 53 (38) | 55 (39) |  | 23 (16) | 48 (33) |  |
| No | 88 (62) | 86 (61) |  | 114 (79) | 86 (60) |  |
| Unknown | 0 | 0 |  | 7 (5) | 10 (7) |  |
| GvHD prophylaxis |  |  | 0.090 |  |  | 0.715 |
| Tac + MMF ± others | 24 (17) | 32 (23) |  | 16 (11) | 18 (13) |  |
| Tac + MTX ± others | 100 (71) | 82 (58) |  | 128 (89) | 126 (88) |  |
| CSA + MMF ± others | 4 (3) | 11 (8) |  | 0 | 0 |  |
| CSA + MTX ± others | 13 (9) | 16 (11) |  | 0 | 0 |  |
| Use of ATG or Campath |  |  | 1.000 |  |  | 1.000 |
| No ATG or CAMPATH | 141 (100) | 141 (100) |  | 144 (100) | 144 (100) |  |
| Year of Transplant |  |  | 0.585 |  |  | 0.173 |
| 2002 | 0 | 1 (1) |  | 0 | 1 (1) |  |
| 2003 | 5 (4) | 4 (3) |  | 0 | 0 |  |
| 2004 | 9 (6) | 12 (9) |  | 3 (2) | 4 (3) |  |
| 2005 | 10 (7) | 13 (9) |  | 1 (1) | 6 (4) |  |
| 2006 | 8 (6) | 12 (9) |  | 1 (1) | 2 (1) |  |
| 2007 | 13 (9) | 17 (12) |  | 6 (4) | 2 (1) |  |
| 2008 | 17 (12) | 12 (9) |  | 10 (7) | 10 (7) |  |
| 2009 | 13 (9) | 19 (13) |  | 12 (8) | 14 (10) |  |
| 2010 | 23 (16) | 11 (8) |  | 13 (9) | 10 (7) |  |
| 2011 | 5 (4) | 6 (4) |  | 13 (9) | 22 (15) |  |
| 2012 | 7 (5) | 4 (3) |  | 25 (17) | 14 (10) |  |
| 2013 | 10 (7) | 13 (9) |  | 19 (13) | 19 (13) |  |
| 2014 | 9 (6) | 9 (6) |  | 21 (15) | 14 (10) |  |
| 2015 | 11 (8) | 8 (6) |  | 13 (9) | 15 (10) |  |
| 2016 | 1 (1) | 0 |  | 5 (3) | 11 (8) |  |
| 2017 | 0 | 0 |  | 2 (1) | 0 |  |

^a^ The Pearson chi-square test was used for comparing discrete variables; the Kruskal-Wallis test was used for comparing continuous variables.
